# Breadth or conservation score (CS): which is more important for HIV-1 T cell based vaccine immunogen design?

**DOI:** 10.1186/1742-4690-9-S2-P260

**Published:** 2012-09-13

**Authors:** P Kunwar, NR Hawkins, X Yu, Y Liu, A Collier, EE Gabriel, T Hertz, H Horton

**Affiliations:** 1 University of Washington/Seattle Biomedical Research Institute, Seattle, WA, USA; 2 Statistical Center for HIV/AIDS Research & Prevention (SCHARP), Seattle, WA, USA; 3 Department of Microbiology, University of Washington, Seattle, WA, USA; 4 Department of Medicine, University of Washington, Seattle, WA, USA; 5 Seattle Biomedical Research Institute, Seattle, WA, USA

## Background

One of the greatest challenges to develop an efficacious HIV vaccine is the enormous diversity of HIV-1. To tackle this problem, T cells based vaccine approaches have come up with two main camps: the mosaic immunogen camp: increasing the breadth of vaccine-induced responses, and the conserved immunogen camp: targeting vaccine-induced T-cell responses only to highly conserved viral regions. While both approaches are theoretically sound, there is no current data suggesting that either approach will be successful in inducing T cells with superior antiviral efficacy. Here we analyzed T cell responses elicited during early HIV-1 infection, to address the question whether CS of targeted epitopes and breadth of T cell responses play an important role in viral control.

## Methods

Using IFN-γ ELISpot, we comprehensively mapped T cell epitope specificities recognized by 24 ART-naïve individuals during early infection. We identified CS of targeted epitopes, where the CS is defined as the proportion of random HIV-1 group M amino acid sequences in the LANL database that include the epitope. We used a prediction model to impute the viral load (VL) set-point using the first available VL as a predictor for subjects lacking VL set-point. We further evaluated the association between the CS of the targeted epitopes and breadth of T cell responses to the individuals’ VL set-point.

## Results

The breadth of CD8+ T cell responses inversely correlated with VL set-point (r=-0.46, p=0.025). Subjects possessing CD8+ T cells recognizing at least one conserved epitope had a lower VL set-point compared to those recognizing only variable epitopes (p=0.093).

## Conclusion

Breadth and CS of HIV-specific CD8+ T cells elicited during early infection are both important for controlling viral replication in vivo. Rationale design of immunization approaches should aim at eliciting a greater breadth of CD8+ T cell to conserved epitopes.

This project is funded by NIH grant#R01AI090783.

